# Comparing adaptive coding of reward in bipolar I disorder and schizophrenia

**DOI:** 10.1002/hbm.26078

**Published:** 2022-09-16

**Authors:** Mariia Kaliuzhna, Matthias Kirschner, Philippe N. Tobler, Stefan Kaiser

**Affiliations:** ^1^ Clinical and Experimental Psychopathology Group, Department of Psychiatry University of Geneva Geneva Switzerland; ^2^ Montreal Neurological Institute McGill University Montreal Canada; ^3^ Laboratory for Social and Neural Systems Research, Department of Economics University of Zurich Zurich Switzerland; ^4^ Department of Psychiatry, Psychotherapy and Psychosomatics Psychiatric Hospital, University of Zurich Zurich Switzerland

**Keywords:** adaptive coding, bipolar disorder, fMRI, monetary incentive delay task, reward processing, schizophrenia

## Abstract

Deficits in neural processing of reward have been described in both bipolar disorder (BD) and schizophrenia (SZ), but it remains unclear to what extent these deficits are caused by similar mechanisms. Efficient reward processing relies on adaptive coding which allows representing large input spans by limited neuronal encoding ranges. Deficits in adaptive coding of reward have previously been observed across the SZ spectrum and correlated with total symptom severity. In the present work, we sought to establish whether adaptive coding is similarly affected in patients with BD. Twenty‐five patients with BD, 27 patients with SZ and 25 healthy controls performed a variant of the Monetary Incentive Delay task during functional magnetic resonance imaging in two reward range conditions. Adaptive coding was impaired in the posterior part of the right caudate in BD and SZ (trend level). In contrast, BD did not show impaired adaptive coding in the anterior caudate and right precentral gyrus/insula, where SZ showed deficits compared to healthy controls. BD patients show adaptive coding deficits that are similar to those observed in SZ in the right posterior caudate. Adaptive coding in BD appeared more preserved as compared to SZ participants especially in the more anterior part of the right caudate and to a lesser extent also in the right precentral gyrus. Thus, dysfunctional adaptive coding could constitute a fundamental deficit in severe mental illnesses that extends beyond the SZ spectrum.

## Graphical abstract

Patients with schizophrenia show reduced adaptation to reward context, a feature that could play a role in the symptoms of apathy. We show that patients with Bipolar disorder also show a deficit in adaptive coding of reward.

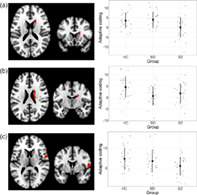

## 1 INTRODUCTION

Recent dimensional approaches to psychopathology highlight the continuity between schizophrenia (SZ) and bipolar disorder (BD) at different levels including clinical presentation, brain morphology, genetic markers and brain connectivity (Yamada et al., 2020). Alterations in reward processing have been described in both disorders, but previous literature remains inconclusive concerning continuity in reward processing deficits between the two disorders (Kirschner, Cathomas, et al., 2020; Kirschner, Rabinowitz, et al., 2020; Nielsen et al., 2012; Schreiter et al., 2016). While SZ findings converge towards reduced striatal activation during the processing of reward, this result is less clear in BD. Thus, depending on the task, task stage (anticipation of reward or reward outcome) and patients' clinical state (depressed, euthymic, manic) increased (Dutra et al., 2015; Mason et al., 2014; Nusslock et al., 2012), decreased (Abler et al., 2008; Redlich et al., 2015; Schreiter et al., 2016; Schwarz et al., 2020; Trost et al., 2014) and similar (Chase et al., 2013; Kirschner, Cathomas, et al., 2020; Smucny et al., 2021) brain responses as compared to control participants have been observed in BD.

**FIGURE 1:**
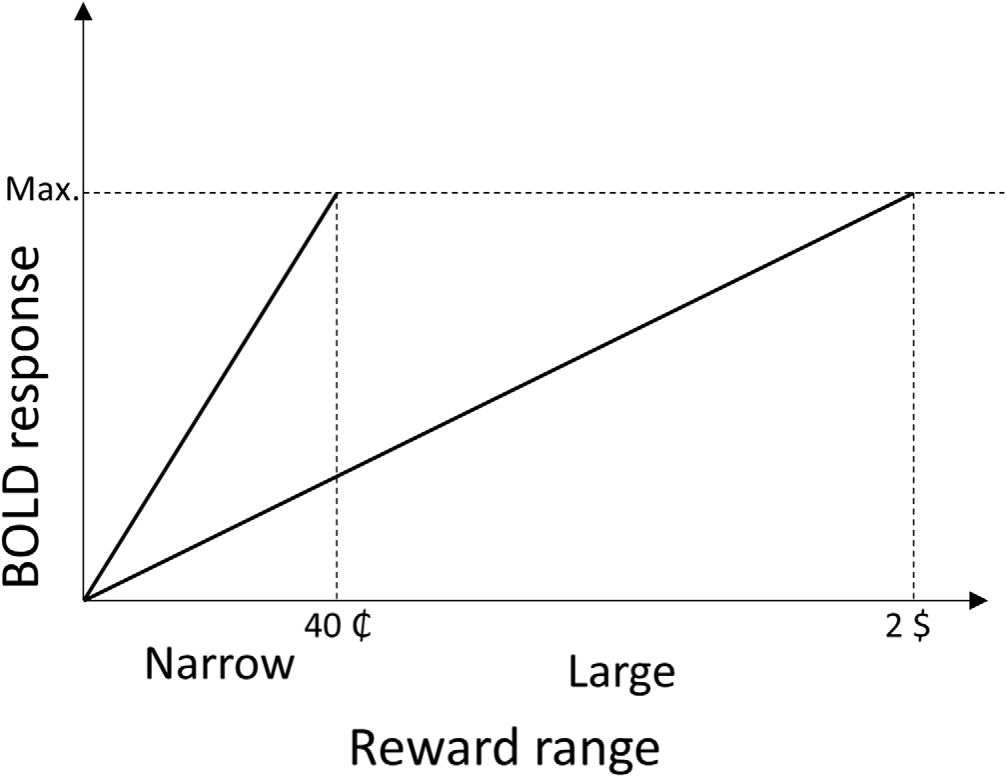
Schematic representation of adaptive reward coding during the MID task, as observed in the caudate of healthy controls. Efficient encoding of all possible rewards with a limited coding range requires the brain to dynamically adapt the response sensitivity to the currently available reward range. Accordingly, a more shallow slope of the BOLD response is expected (and observed) in the large reward range condition (2$) than in the small reward range condition (40₵). By extension, the same reward (e.g., 40₵) will elicit different responses depending on the context it is presented in: A maximal response in the small reward range and an intermediate response in the large reward range condition

One of the core mechanisms underlying efficient reward processing in healthy participants is adaptive coding. Adaptive coding is the ability of neurons to adjust their response according to the present context (Kourtzi & Connor, 2011; Smirnakis et al., 1997). Extensively described in perception, adaptive coding is also characteristic of midbrain dopaminergic neurons, which, rather than coding the absolute value of a received reward, adapt their responses to the range of most probable rewards at a given time (Tobler et al., 2005). Physiological studies in non‐human primates suggest that range adaptation allows for a more precise encoding of the stimulus, by making optimal use of the entire firing range of the neuron. In humans, several reward‐sensitive regions, such as the striatum, have been shown to adapt their response depending on the reward context (Burke et al., 2016). Specifically, when the range of possible rewards is narrow (e.g., you can win up to 40 cents) these regions show a steeper response curve, that is, the BOLD response increases more strongly to increasing reward amounts received by healthy participants (Figure 1) (Kirschner et al., 2016; Kirschner et al., 2018). Conversely, when the range of possible outcomes is wide (e.g., you can win up to 2$) and more potential rewards need to be encoded, the increase in the BOLD response to increasing reward amounts is shallower.

Deficient adaptive coding of reward in the caudate and the insula/precentral gyrus has been described for the whole SZ spectrum, including participants with schizotypal personality traits, patients with first‐episode psychosis, as well as patients with SZ (Kirschner et al., 2016; Kirschner et al., 2018). These participants show similar response increases for wide and narrow reward ranges, indicating reduced contextual influence. Although a dopamine dysfunction affects both SZ and BD (Jauhar et al., 2017), nothing is known about whether patients with BD can represent reward in an adaptive fashion.

In the present work, we sought to establish whether adaptive coding of reward is reduced in euthymic patients with BD similarly to patients with SZ.

Here, we used the same paradigm and analysis approach as in our previous work on adaptive coding of reward across the SZ spectrum (Kirschner et al., 2016; Kirschner et al., 2018). Combining a version of the monetary incentive delay task with fMRI, we compare adaptive coding between three groups: BD patients, SZ patients and healthy controls. We sought to establish whether adaptive coding deficits are present in BD to a similar extent and in the same brain regions as in SZ, and, if the deficits, should they exist, relate to shared symptoms of BD and SZ.

## 2 MATERIALS AND METHODS

### 2.1 Participants

Twenty‐five patients with BD‐I participated in the study; 18 outpatients and 7 inpatients at the end of hospitalization (one patient was excluded from the analysis due to corrupt data files). Data from patients with SZ and healthy controls have previously been reported by Kirschner et al. (2016), and included 27 patients with SZ (16 inpatients and 11 outpatients) and 25 healthy controls (HC) (Table 1). Data from all participants were collected in the same time period and no software or hardware changes have been introduced in between. In addition, the interviews for rating psychopathology were conducted by the same researchers for all the groups.

**TABLE 1:** Socio‐demographic and neuropsychological characteristics of the participants

	BD‐I (*N* = 25)	SZ (*n* = 27)	HC (*n* = 25)	BD‐I vs. HC BD‐I vs. SZ	BD‐I vs. SZ
Age (year)	37.3 ± 9.1	31.9 ± 7.4	33.1 ± 9.7	*t* = −1.579, *p* = .121	*t* = −2.343, *p* = .023
Sex (male/female)	16/9	18/9	16/9	*χ* ^2^ = 0, *p* = 1	*χ* ^2^ = 0.041, *p* = .840
Education (year)	14.6 ± 3.5	12.2 ± 3.1	12.4 ± 3.6	*U* = 194.5, *p* = .021	*U* = 190.5, *p* = .007
Duration of illness (year)	15.4 ± 9.0	9.2 ± 6.6			*U* = 197.5, *p* = .01
Age of onset (year)	21.9 ± 5.7	22.7 ± 6.0			*t* = 0.472, *p* = .639
Handedness (*r*/l)	24/1	24/3	22/3	χ^2^ = 1.087, *p* = .297	χ^2^ = 0.924, *p* = .336
Psychopathology					
*BNSS score*					
Apathy (motivation and pleasure)	13.0 ± 8.3	14.8 ± 6.9			*t* = 0.839, *p* = .406
Diminished expression	4.32 ± 4.5	8.4 ± 7.2			*U* = 224, *p* = .037
BNSS total score	18.1 ± 12.1	24.6 ± 12.4			*U* = 238, *p* = .068
*PANSS score*					
Positive (P1, P3, P5, G9)	4.4 ± 1.0	6.6 ± 2.5			*U* = 149.5, *p* < .0001
Negative (N1, N2, N3, N4, N6, G7)	10.3 ± 3.7	13.6 ± 5.2			*U* = 213, *p* = .022
Disorganized (P2, G5, N11)	3.6 ± 1	4.5 ± 2.2			*U* = 276.5, *p* = .212
Excited (P4, P7, G8, G14)	4.3 ± 0.6	5.1 ± 1.5			*U* = 209.5, *p* = .007
Depressed (G2, G3, G6)	5.5 ± 1.6	5.1 ± 2.2			*U* = 274, *p* = .237
Total PANSS score	40.0 ± 5.6	49.4 ± 11.2			*t* = 3.906, *p* < .001
HAMD 21 total score	4.7 ± 4.2	2.0 ± 4.2			*U* = 157.5, *p* < .0001
CDSS total score	3.4 ± 3.0	1.6 ± 2.2			*U* = 204, *p* = .012
GAF score	64.8 ± 12.1	56.9 ± 9.6			*t* = −2.613, *p* = .012
PSP total score	63.9 ± 14.4	56.4 ± 9.9			*t* = −2.163, *p* = .036
Cognition					
Composite Cognitive Ability	−0.256 ± 0.8	−0.616 ± 0.9	0 ± 0.5	*t* = 1.262, *p* = .215	*t* = −1.452, *p* = .153
MWT IQ	27.6 ± 3.9	25.9 ± 5.8	27.6 ± 4.0	*t* = 0.030, *p* = .976	*t* = −1.155, *p* = .254

*Note*: BNSS Apathy was defined based on the BNSS subscales anhedonia (items 1–3), asociality (items 5, 6) and avolition (items 7, 8) and BNSS Diminished expression was defined as the sum of the BNSS subscales blunted affect (items 9–11) and alogia (items 12, 13); Data are presented as average ± SD.

Abbreviations: BD‐I, bipolar disorder I; BNSS, Brief Negative Symptom Scale; CDSS, Calgary Depression Scale for Schizophrenia; GAF, Global Assessment of Functioning; HAMD, Hamilton Depression Rating Scale; MWT IQ, Multiple Word Test Intelligence Quotient; PANSS, Positive and Negative Syndrome Scale; PSP, Personal and Social Performance Scale; SZ, schizophrenia.

All participants were between 18 and 55 years old. The diagnosis of BD‐I and SZ was confirmed using the Mini‐International Neuropsychiatric Interview for DSM‐IV (MINI). We excluded patients with schizoaffective disorder, current major depressive, manic or hypomanic episode, as well as patients with any other DSM‐IV axis I disorder or neurological disorder. All patients were clinically stable with no change to medication for at least 2 weeks prior to inclusion in the study. The absence of major extrapyramidal symptoms was confirmed using the Modified Simpson‐Angus Scale (total score ≤2). BD‐I patients were clinically euthymic and did not present with either more than sub‐syndromal depressive symptoms (Hamilton Depression Rating Scale [HAMD] score < 17), as defined by the International Society for Bipolar Disorder Task Force (Tohen et al., 2009) or manic symptoms (confirmed using the Young Mania Rating Scale (Young et al., 1978), mean = 0.4, *SD* = 0.91, min = 0, max = 4). In HC, the MINI was used to confirm the absence of any current or previous neurological or psychiatric conditions. The study was approved by the local ethics committee of the canton of Zurich and all participants provided written informed consent.

To clinically assess depressive symptoms, we used the Hamilton Depression Rating Scale (HAMD 21 (Hamilton, 1967)) and the Calgary Depression Scale for SZ (CDSS, (Addington et al., 1990)). Negative symptoms were assessed using the Brief Negative Symptom Scale (BNSS, (Kirkpatrick et al., 2011)) and factor scores for apathy and diminished expression were calculated according to Mucci et al (Mucci et al., 2015). To assess the general level of functioning, we used the Global Assessment of Functioning Scale (GAF, (Frances et al., 1994)) and the Personal and Social Performance Scale (PSP, (Juckel et al., 2008)). Psychotic symptoms were assessed using the Positive and Negative Syndrome Scale (PANSS, (Kay et al., 1989). Cognition was assessed using the Brief Neurocognitive Assessment (Fervaha et al., 2015)).

### 2.2 Procedure

Participants performed a variant of the Monetary Incentive Daly Task (MID), originally developed by Simon and colleagues (Simon et al., 2015), that uses stimuli based on the Cued‐Reinforcement Reaction Time Task (Cools et al., 2005). Before the beginning of the task, participants were informed that they would receive all the money they would win during the experiment. At the start of every trial one of three cues, signalling no reward, small reward, or large reward, indicated the reward context. In the no‐reward condition, participants won nothing. In the small reward condition, they could win between 0 and 40 cents, and in the large reward condition, they could win between 0 and 2 Swiss francs. The exact amount won depended on participants' speed (RT on the current trial compared to RTs in the previous 15 trials) and accuracy (incorrect or late [>1 s] response resulted in a reward of 0). The response was elicited by three circular shapes presented after the cue and the participants had to indicate the odd one out (right or left button press), responding as fast and as correctly as possible. Participants were then given feedback as to the amount won on the trial. The maximal amount that could be won at the end of the experiment was 50 Swiss francs. Two training sessions (10 trials each) were conducted—one outside and one inside the scanner. The experimental session consisted of two runs, 36 trials each. The duration of every trial was ~10 s: cue (0.75 s), ISI (2.5–3 s), target (1 s max), outcome (2 s). The inter‐trial interval was jittered from 1 to 9 s with a mean of 3.5 s. Each run lasted about 6 min.

### 2.3 Functional imaging acquisition

Imaging data were collected using a Philips Achieva 3.0 T magnetic resonance scanner with a 32‐channel SENSE head coil at the MR Centre of the Psychiatric Hospital, University of Zurich. Functional MRI scans were acquired in 2 runs with 195 images in each run. A gradient‐echo T2*weighted echo‐planar image (EPI) sequence with 38 slices acquired in ascending order was used. Acquired in‐plane resolution was 3 × 3 mm^2^, 3 mm slice thickness and 0.5 mm gap width over a field of view of 240 × 240 mm^2^, repetition time 2000 ms, echo time 25 ms and flip angle 82°. Slices were aligned with the anterior–posterior commissure. Anatomic data were acquired using an ultrafast gradient‐echo T1‐weighted sequence in 160 sagittal plane slices of 240 × 240 mm^2^ resulting in 1 × 1 × 1 mm voxels.

### 2.4 Data analysis

#### 2.4.1 Data analysis followed the same pipeline as in our previous work (Kirschner et al., 2018)

Functional magnetic resonance imaging data were analysed using SPM8 (Statistical Parametric Mapping, Wellcome Department of Cognitive Neurology, London, UK). Statistical analyses were performed using R (R Core Team, 2022).

### 2.5 Behavioural data analysis

Reaction times to the target were the main outcome measure of the MID task. A two‐way repeated measures ANOVA was conducted on RTs, with group as between subject factor and the reward condition (no, small and large reward) as within subject factor. Bonferroni‐corrected pairwise comparisons were calculated as post hoc tests for significant main effects. One BD‐I subject was excluded only from the behavioural analysis due to corrupted data files.

### 2.6 Image preprocessing

Data were preprocessed as described in Kirschner et al., 2016, 2018 (see Supplemental material). To assure adequate quality of fMRI data motion and susceptibility artifacts were detected using the Art toolbox (http://web.mit.edu/swg/software.htm) and detected outlier scans were replaced by the mean image of the session for each participant. No subjects were removed from the analysis as a result of this procedure.

### 2.7 First‐level image analyses (within subject)

Following Kirschner et al. (2018), in a first step, we used a general linear model with a parametric design to identify brain regions encoding the amount of reward obtained during the outcome phase. For this, each reward outcome condition (no‐, small‐ and large reward) was modelled separately. The three outcome regressors accounted for the mean activations in the three conditions and allowed us to assess potential effects of group on small (0–0.40 CHF) and large (0–2 CHF) reward outcomes in general. Importantly, the small and large outcome regressors were parametrically modulated by the outcome received by each participant in each trial during the experiment (pmod small reward and pmod large reward respectively). The two parametric modulators capture linear deviations from the mean activity induced by the trial‐specific reward level and are orthogonal to the mean regressors. The following regressors of no interest were used: one regressor for the anticipation phase (duration between 3.25 and 3.75 s), one regressor for target presentation, and, finally, one regressor for trials where participants made an error (modelled at target presentation). Thus, eight regressors were used for the first level analysis. All the explanatory variables were convolved using the canonical haemodynamic response function. We note that the two parametrically modulated reward regressors of interest were not correlated with the anticipation regressor, the latter serving to account for unspecific visual activations caused by stimulus presentation during the anticipation phase.

### 2.8 Second‐level image analyses (group comparison)

At the second (i.e., group) level, we interrogated the individual contrast images from the first‐level parametric modulators in a two‐step procedure.

First, we identified brain regions processing the reward amount received in the outcome phase, by assessing the contrast (pmod small reward + pmod large reward) in a voxel‐wise whole‐brain analysis across all participants with a one‐tailed *t*‐test. The statistical threshold was set to *p* < .05, whole‐brain voxel‐level family‐wise error (FWE) rate corrected for multiple comparisons. In the second step, we investigated adaptive coding of reward across the three groups. The following adaptive coding contrast was run within the regions of interest (ROIs) identified in step 1: the contrast estimates of the large reward parametric regressor were subtracted from the contrast estimates for the small reward parametric regressor (pmod small reward − pmod large reward). A one‐tailed voxel‐wise *t*‐test was used.

To investigate between‐group differences, we extracted the mean contrast estimates of the adaptive coding contrast obtained in step 2 (pmod small reward − pmod large reward) from the reward‐sensitive regions obtained in step 1 (pmod low reward + pmod high reward) with the Marsbar toolbox. We then compared the three groups using Fischer's one‐way ANOVAs for each region and Tukey post hoc comparisons.

### 2.9 Correlational analyses

To test for any associations between different symptoms and the adaptive coding contrast estimates in the BD group, we performed two‐tailed Spearman rank correlation or Pearson correlation analyses within the regions showing significant group differences.

## 3 RESULTS

### 3.1 Demographic and clinical data

Group characteristics are summarized in Table 1. Compared to SZ participants, BD participants were older, had longer illness duration, more years of education, as well as a higher level of functioning (as indicated by higher GAF and PSP scores). Eighteen out of 25 BD participants were treated with atypical antipsychotics; 18 participants were prescribed mood stabilizers and 7 participants received antidepressants. All SZ participants were treated with atypical antipsychotics. Mean chlorpromazine equivalents were lower in the BD group compared to the SZ group (BD‐I: 185.99 ± 259.8, SZ: 508.01 ± 369.2; *U* = 133, *p* < .001). None of the patients received typical antipsychotics.

### 3.2 Behavioural results

Response time (RT) was the main measure of task performance. An ANOVA on the RTs in the three conditions showed no significant effect of group (*F*[2, 73] = 1.54, *p* = .22) nor condition * group interaction (*F*[4, 142] = 1.92, *p* = .11), but, as in our previous studies, a significant effect of reward (*F*[2,73] = 65.4, *p* < .001). Specifically, larger rewards were associated with faster RTs. Furthermore, one way ANOVAs revealed no group differences for total accuracy (*F*[2, 47.1] = 2.25, *p* = .12) but for total amount won during the task (*F*[2, 48.2] = 4.09, *p* = .023). Tukey post hoc tests showed that participants with BD‐I won more than participants with SZ (*p* = .03) and the amounts won did not differ between BD‐I and HC (*p* = .9). Overall, these results show that all groups were able to perform the task.

### 3.3 Adaptive coding of reward: Group analysis

In the first step, brain areas processing reward amount were identified in a voxel‐wise whole‐brain analysis across the three groups of participants. The identified regions showed increased activation with increasing reward during the outcome phase of the experiment. The results mirror the activations reported in our previous work (Table 2), as well as other studies on reward processing, and show significant effects in the right caudate and the right precentral region.

**TABLE 2:** Whole‐brain analyses of reward coding regions across all participants

	X	Y	Z	Cluster size	*t*
Right precentral gyrus	60	3	12	250	6.77
	55.5	−3	7.5		4
Right posterior caudate	21	−4.5	27	574	5.43
	19.5	−15	24		5.30
	25.5	−31.5	28.5		5.26
Right anterior caudate	16.5	19.5	16.5	145	5.12
Left middle frontal gyrus	−24	22.5	54	2957	6.65
	−16.5	18	61.5		6.28
	−16.5	33	54		5.95
Left angular gyrus	−48	−60	40.5	1814	6.09
	−31.5	−61.5	24		5.15
	−57	−39	42		4.96
mOFC	−7.5	54	−6	456	6.08
	−1.5	63	−1.5		4.79
	−1.5	57	9		4.04
Right superior occipital gyrus	15	−88.5	24	1582	6.00
	15	−93	13.5		5.72
	3	−75	22.5		5.17
Left orbitofrontal cortex	−9	24	−16.5	1443	5.75
	−19.5	4.5	−12		5.68
	−13.5	9	−18		5.35
Left postcentral gyrus	−21	−31.5	61.5	406	5.15
	−21	−33	76.5		4.52
	−28.5	−28.5	66		4.19
Right postcentral gyrus	28.5	−40.5	63	301	5.13
	27	−45	72		4.44
	31.5	−39	55.5		4.42
Left caudate	−19.5	−3	27	471	5.49
	−18	6	24		4.43
	−21	13.5	22.5		4.3
Right middle frontal gyrus	30	33	13.5	486	5.04
	9	30	4.5		4.56
	−3	27	6		4.56
Right putamen	22.5	6	−10.5	492	4.95
	22.5	25.5	−6		4.72
	24	16.5	−9		4.48
Left posterior ventral cingulate cortex	−4.5	−49.5	31.5	977	4.72
	0	−58.5	25.5		4.42
	−4.5	−55.5	16.5		4.18

In step 2, we compared the three groups in a single analysis. A one‐tailed *t*‐test with the adaptive coding contrast in the three groups was run in the ROIs identified in step 1, showing a network of regions responding to reward adaptively (Table 3).

**TABLE 3:** Analysis of adaptive coding of reward across all participants

	X	Y	Z	Cluster size	t
Right precentral gyrus	60	3	12	175	5.99
Right caudate	19.5	−15	24	157	4.48
	21	−6	30		3.97
	19.5	3	22.5		3.70
Left postcentral gyrus	−19.5	−31.5	60	152	5.14
	−22.5	−30	67.5		3.47
Left precentral gyrus	−33	−15	55.5	107	4.85
Left middle fontal gyrus	−24	22.5	54	221	4.59
	−16.5	33	54		4.52
Right paracentral lobule	4.5	−27	61.5	157	4.46
	3	−39	60		3.62
Right superior occipital gyrus	15	−88.5	22.5	159	4.25
	16.5	−94.5	9		4.15
	22.5	−90	12		3.40
Left caudate	−21	−6	25.5	144	4.06
	−18	6	24		3.58

To test for group differences in adaptive coding, we applied a cluster defining threshold *p* = .0005, FWE peak‐level correction *p* < .05 to the pmod small reward + pmod large reward contrast, so as to obtain clusters that were anatomically more constrained. Sixteen regions were identified (Table S1). We then ran a one‐tailed *t*‐test with the adaptive coding contrast (pmod small reward − pmod large reward) in these 16 ROIs, extracted the activation using the Marsbar toolbox and compared them across groups using Fisher's one‐way ANOVA (Table S2). Tuckey post hoc tests were then run to compare between groups.

**FIGURE 2:**
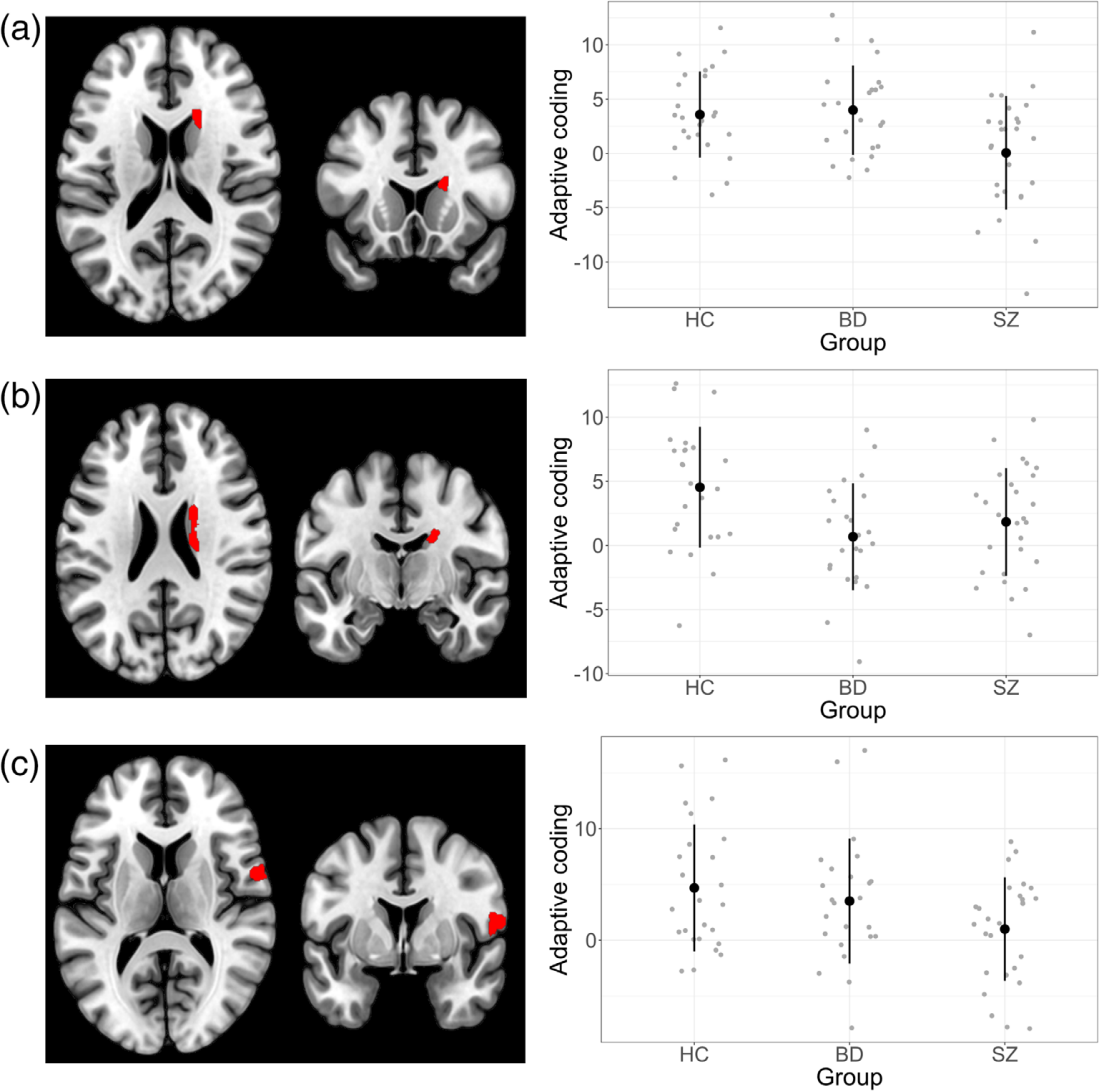
Reward‐sensitive regions showing group differences in adaptive coding. Left panels: Reward‐sensitive regions ([pmod small reward] + [pmod large reward]) used as regions of interest (ROI) for the adaptive coding contrast ([pmod small reward] − [pmod large reward; right panels]). (a) Anterior right caudate. (b) More posterior right caudate. (c) Right precentral gyrus

A significant result was obtained for 4 regions. Two regions of the right caudate showed a significant group difference (Figure 2). In the more anterior region (*F*[2,73] = 6.03, *p* = .004), the SZ group showed significantly weaker adaptive coding than the HC (*p* = .02) and BD groups (*p* = .007), while the BD group was at the same level as HC (*p* = .9). In the more posterior region (*F*[2,73] = 5.1, *p* = .008), the SZ group was not significantly lower than the HC group (*p* = .072), but the BD group was (*p* = .008). There was no significant difference between the two patient groups (*p* = .6). Thus, while patient with SZ show impaired adaptive coding in both caudate subregions (at the trend level in the posterior part), BD patients showed impaired adaptive coding only in the posterior caudate.

In addition, a significant group difference was also observed also for the right precentral gyrus BA 6/insula (*F*[2,73] = 3.3, *p* = .043). As in our previous results, the SZ group showed significantly weaker adaptive coding compared to the HC group (*p* = .038). Patients with BD, on the other hand, were not different from either the HC group (*p* = .7), or the SZ group (*p* = .2). This suggests that in the precentral/insular region adaptive coding in BD patients is situated between HC and patients with SZ.

Finally, a significant result was also observed for the right superior occipital gyrus/cuneus (*F*[2,73] = 6.16, *p* = .003). Both BD‐I and SZ patients showed reduced adaptive coding with respect to controls (BD‐I *p* = .005 and SZ *p* = .02), with no difference between the two patient groups (*p* = .8). There were no significant results for the other regions (Table S2).

### 3.4 Comparing responses to small and large rewards (pmod high vs. pmod low)

**FIGURE 3:**
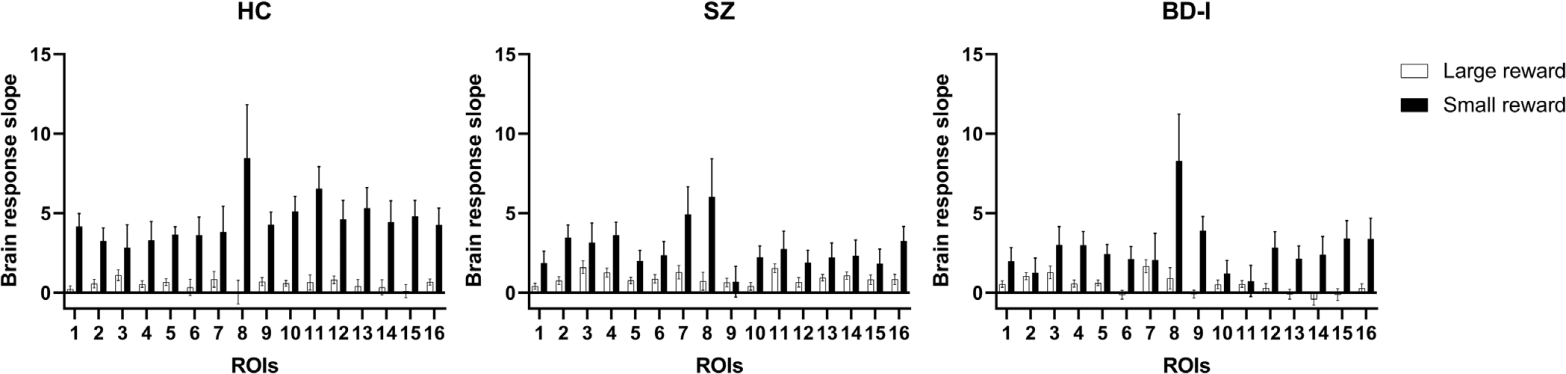
Results of the pmod small reward (black bars) and pmod large reward contrasts (white bars) for the three groups of participants in the 16 brain regions identified by the (pmod small reward) + (pmod large reward) contrast. As expected, a steeper increase in the brain response is elicited in the small reward context, than in the large reward context. Error bars represent SE. The numbers represent the following ROIs: 1. Left caudate 2. Left angular gyrus 3. Left anterior prefrontal cortex 4. Left orbitofrontal cortex 5. Left middle frontal gyrus 6. Left postcentral gyrus 7. Left posterior ventral cingulate cortex 8. Left thalamus 9. Right anterior caudate 10. Right posterior caudate 11. Right superior occipital gyrus/cuneus 12. Right middle frontal gyrus 13. Right primary motor area 14. Right postcentral gyrus 15. Right precentral gyrus 16. Right putamen

Patients' deficit of adaptive coding in these regions could be due to an abnormal brain response to either small rewards or large rewards, or both. To establish the origin of this deficit, we ran a repeated measures ANOVA comparing brain activation elicited by small rewards (pmod small) to activation elicited by large rewards (pmod large) between patients and controls (Figure 3), in the four above regions where a reduction in adaptive coding was observed in patients. The ANOVA yielded a significant 3‐way interaction between Group, Reward magnitude (small, large) and brain region (*F*[3, 6] = 2.6, *p* = .016). Importantly, no differences were found between groups for large rewards in either brain region (all *p* > .99). That is, the increase in the brain response to increasing rewards in the large reward context was comparable between groups.

Conversely, for small rewards, SZ patients had significantly shallower slopes than HC in the anterior part of the right caudate (*p* = .03), whereas patients with BD were not different from either HC (*p* = .9) or SZ (*p* = .11). Furthermore, for small rewards, BD patients showed significantly shallower slopes than HC in the posterior part of the right caudate (*p* = .003) and the right cuneus (*p* = .002). In these regions SZ patients did not differ from either HC (all *p* > .12) or BD (all *p* = .9). Thus, SZ and BD‐I patients had a weaker increase in brain response to increasing rewards in the narrow range, where a steeper increase in brain response is usually observed.

Comparing between small and large rewards within each group yielded the following results. For the HC group, Tukey post hoc tests showed a significant difference between small and large rewards in all the four brain regions (all *p* < .038). For the SZ group, there was no difference between the two conditions in either brain region (all *p* > .87). For the BD‐I group, in the anterior part of the right caudate, there was a significant difference between low and high reward conditions (*p* = .012), but no difference was found in the other brain regions (all *p* > .22). Thus, the deficit in adaptive coding (i.e., difference between brain response in small vs. large reward conditions) observed between the three groups is a result of a shallower increase in brain response to rewards predominantly in the small reward condition.

### 3.5 Correlational analyses in the BD‐I group

In BD, adaptive coding in the more anterior part of the right caudate correlated positively with the total amount won in the task (*r*_p_ = 0.48 *p* = .017).

In terms of symptomatology, a significant negative correlation was observed between adaptive coding in the more posterior section of the right caudate and the general subscale of the PANSS (*r*_s_ = −0.45 *p* = .029). No correlations between adaptive coding and other psychopathology measures (HAMD, CSDS, YMRS, PANSS positive or total subscales) were found.

In addition, a trend‐level positive correlation was observed between adaptive coding in the right precentral gyrus and chlorpromazine equivalents for antipsychotic medication (*r*_s_ = 0.38, *p* = .065).

For correlational analyses in the HC and SZ groups see Tables S3–S5. In these two groups, the total amount won on the task did not correlate with adaptive coding. In the SZ group, similarly to the results reported in Kirschner et al., 2016, PANSS positive and PANSS total scores correlated negatively with adaptive coding in the right anterior caudate.

## 4 DISCUSSION

The present work explored adaptive coding in patients with BD‐I using a version of the monetary incentive delay task, comparing their brain response to rewards to that of patients with SZ and HC. As in SZ, adaptive coding appears to be impaired in BD‐I. However, we observed several differences between SZ and BD patients. Specifically, adaptive coding was significantly reduced in BD‐I participants in the more posterior part of the right caudate but preserved in its more anterior part, in contrast to patients with SZ. In the right precentral gyrus, BD‐I patients showed intermediate levels of neural adaptation to reward.

Extending our findings from previous work, our present results show that adaptation to reward occurs in multiple areas of a reward encoding network (Figure 3) in HC but also in both patient groups. Intact reward adaptation in multiple brain areas might explain that both SZ and BD‐I patients performed the task very similar to control participants and all differences between groups (e.g., smaller amount won for SZ patients) were small. Crucially, regions central to reward processing did show impaired adaptation in patients.

Similar to patients with SZ, BD‐I patients showed adaptive coding deficits in the more posterior part of the right caudate (in SZ adaptive coding was reduced in this region, at the trend level, with no difference between the two patient groups), a region that underlies reward‐guided action selection and reward learning. In BD, reduced activation in the right caudate during reward feedback has previously been reported in a card‐guessing paradigm (Redlich et al., 2015) for patients who were experiencing a depressive episode. Our previous work in SZ participants has shown adaptive coding in the caudate to be negatively associated with increased total symptom severity, as measured by the PANSS, but also its general and negative subscales. Similar results for SZ patients were obtained in the present analysis, see Table S4 (though some associations were trend‐level). In BD patients, we observed a negative association between adaptive coding in the posterior part of the right caudate (where deficient adaptive coding was observed in this group) and the PANSS general subscale. Thus, in both disorders adaptive coding may represent a general deficit, spanning multiple areas of motivation, perception and cognition. Indeed, dopamine neurons send widespread projections across the brain and their insensitivity to context might underlie a broad range of deficits. In BD, no associations were found between specifically depressive (as measured by the HAMD), manic or psychotic symptoms and adaptive coding (Table S5).

Interestingly, in the BD group, adaptive coding in the more anterior part of the right caudate (where adaptive coding appeared intact in this group) correlated positively with the amount won on the task, that is, the stronger the adaptive coding, the more participants won. Previous work has indeed shown an association between striatal activity and reward task performance in healthy participants (Chib et al., 2012; Mobbs et al., 2009; Schonberg et al., 2007; Watanabe et al., 2019), although such an association has not, to our knowledge, been reported for MID tasks. For example, better reward learning has been associated with increased striatal activity. Here, we only find this association in BD patients. As BD patients won more than SZ patients, and at the same level as HC, we can speculate that to the extent that adaptive coding occurred in the anterior right caudate, it rescued performance in this group. It is possible that we do not observe a correlation between striatal activity and performance in the HC group, as they were at ceiling, thus, performance depended less on the activity in an individual brain area. Similarly, previous work has shown correlations between striatal activity and performance for healthy participants who succeeded on a reward learning task, but not in those who failed to learn. Thus, similar to the prediction error signalling in reward learning tasks, adaptive coding in the striatum might distinguish between patient populations who behaviourally show better reward‐related performance and those who do not. However, this speculation is to be taken with caution due to the small sample size and small differences between groups.

Moreover, our findings seem to imply a segregation of the adaptive coding deficit between BD and SZ in the right caudate. Potentially, this deficit segregation between the two conditions might relate to a functional segregation of the caudate. Previous work in healthy participants has indeed indicated that the more anterior part of the caudate is engaged in learning new rules and more cognitive and emotional processing, whereas the more posterior part is dedicated to action‐based processes (Brovelli et al., 2011; Hampshire et al., 2019; Mattfeld & Stark, 2011; Robinson et al., 2012). Thus, SZ patients would not only demonstrate reduced adaptation at the level of the anterior part of the caudate, but also have deficits in selecting the appropriate action in response to rewarding stimuli (Barch et al., 2015; Morris et al., 2015; Morris et al., 2018). On the other hand, BD patients show intact reward adaptation in the anterior caudate, but, instead, might have stronger deficits in action selection and monitoring, in line with previous findings (Chandler et al., 2009; Ibanez et al., 2012).

As in our previous work, SZ patients showed an adaptive coding deficit in the right precentral gyrus/insula. BD participants showed no difference from HC in this region. However, they were also not significantly different from SZ participants, in line with intermediate levels of adaptive coding. In addition to the functional results described above, previous anatomical studies have shown a decrease in grey matter volume in BD patients in the right caudate and right as well as left precentral gyri (Lisy et al., 2011; Lyoo et al., 2004). Of note, adaptive coding in the right precentral gyrus in BD positively correlated at the trend level with the dose of antipsychotic medication they received. We can thus speculate that antipsychotic medication in BD may have a positive effect on context adaptation in this brain region (Liemburg et al., 2012). For the SZ group, no correlations between adaptive coding and antipsychotic medication were found. Including chlorpromazine equivalents as a covariate for the adaptive coding contrast did not yield any significant regions (all *p* > .3).

In order to assess whether BD patients who received antipsychotic medication were more similar to SZ patients, we performed the same group level analyses including, for the BD‐I group, only the 17 patients who were on antipsychotic medication (the results are presented in Supplemental material). The results are almost identical to the ones reported in the manuscript, and do not show more similarity between the two patient groups. We believe several points might explain the weak link between medication and adaptive coding. First, the correlation was only observed for the BD‐I group, and was only at the trend level. No correlation between adaptive coding was observed for the SZ group. SZ patients were also administered over 2.5 times more antipsychotic medication than BD‐I patients (*p* < .001; after removing the patients receiving no antipsychotic treatment the difference between the two groups is still significant – *p* = .026). Thus, BD‐I patients would have had much weaker psychotic symptomatology (as supported by the significantly higher scores in the SZ group on PANSS total, positive, negative and excited subscales, Table 1). It is possible that BD‐I patients' performance would resemble SZ's more if patients were tested in e.g. manic or hypomanic states. Reducing the number of patients in the BD‐I group (from 24 to 17 who took antipsychotic medication) also reduces the power of observing true differences.

Finally, both patient groups showed reduced adaptation in another area associated with reward processing—the right superior occipital gyrus/cuneus (Cao et al., 2019; Mobbs et al., 2009; Morelli et al., 2015; van Hell et al., 2010). Reduced activity in this region in response to reward outcome has previously been reported in BD (Redlich et al., 2015) and SZ (Gradin et al., 2013) patients. Interestingly, hypomania‐prone healthy participants showed increased activity in this region for neutral stimuli, instead of rewarding stimuli, pointing towards contextual blunting (O'Sullivan et al., 2011).

Thus, it appears that both BD and SZ show deficient adaptive coding in several regions. Although this deficit is less pronounced in BD, it could still point towards a continuity in reward processing adaptive coding alterations between the two conditions. Similarly to SZ, a blunted contextual response could be inherent to BD, and the difference between the two conditions could simply be quantitative. Deficient adaptive coding in BD would also be in line with previous research hinting at reduced contextual adaptation related to manic and hypomanic symptoms. Indeed, previous work in healthy participants prone to hypomania has shown a lack of discrimination between stimulus values (O'Sullivan et al., 2011), with neutral outcomes activating the medial temporal lobe similarly to rewards (while controls showed increased activation for rewards). In addition, an earlier study in BD patients performing the MID task during a manic episode found no difference in nucleus accumbens responses induced by receipt and omission of reward at the outcome phase of the task (Abler et al., 2008). Thus, these earlier studies appear to point towards a deficit in contextual adaptation in mania.

In contrast to our findings, some previous work has found no differences between BD patients and HC in reward processing at the outcome phases of different tasks in the striatum (Chase et al., 2013; Johnson et al., 2019; Nusslock et al., 2012; Smucny et al., 2021). It is thus possible that adaptive coding represents a more stringent test of reward processing, beyond simple evaluation of brain activity during the outcome phase of reward tasks. It should also be noted that no significant differences have been observed in brain activity during reward anticipation between the three groups analysed here (Kirschner, Cathomas, et al., 2020). Thus, despite similar behavioural performance and reward anticipation in HC and BD, the latter could still harbour a deficit in adaptation to reward context.

Importantly, we show that the adaptive coding deficit observed in both our patient groups was mostly driven by a shallower increase of the brain response in the small reward condition. When only a narrow range of rewards is available, a steep increase in brain responses to more rewarding stimuli is observed in healthy controls (Cox & Kable, 2014; Kirschner et al., 2016; Kirschner et al., 2018). Patients thus fail to scale up their response to such rewards. In BD‐I, this lack of adaptation to small rewards could underlie impulsive reward seeking: smaller rewards would fail to produce a significant brain response and patients would thus seek stronger, more satisfying rewards. Indeed, a link between reduced nucleus accumbens activity during reward anticipation in remitted BD patients has previously been linked to more impulsive responses to positive emotions (Johnson et al., 2019).

It is also possible that adaptive coding deficits have a different aetiology in BD and SZ. Limitations to transdiagnostic approaches have indeed been voiced, highlighting the fact that, for reward in particular, different mechanisms could lead to similarly altered brain activity patterns (“equifinality”) (Weinberger & Goldberg, 2014; Whitton et al., 2015). In SZ, an adaptive response to reward would be prevented by a simultaneous decrease of adaptive dopamine transients and an increase in spontaneous dopamine transients in the striatum. This would result in a blunted response to rewarding stimuli as well as an increased response to neutral cues (Maia & Frank, 2017), thus, reducing the neural discrimination between the two. In contrast, in BD, the substantial changes in internal state (manic vs. depressive) may lead to a blunted processing of external contexts (O'Sullivan et al., 2011). Although the exact role of dopamine in BD is still a matter of debate, previous work points towards an increase of striatal dopaminergic transmission during mania, and a decrease in the dopaminergic function during depression (Ashok et al., 2017; Cousins et al., 2009). Such extreme fluctuations of the dopaminergic response could lead to its overall blunting, which in turn may result in reduced discriminability of reward stimuli in euthymia, mania and depression. This hypothesis is in line with the interpretation of Redlich and colleagues (2015), who found significantly reduced caudate activation in depressed BD patients compared to patients with unipolar depression (UD). They concluded that BD patients might exhibit a relatively stronger impairment of the mesolimbic structures because they have to regulate the change between manic/hypomanic and depressive states.

Several limitations to the present work should be pointed out. A larger sample size would have allowed to draw more robust conclusions, especially for the right precentral gyrus, where BD patients did not differ significantly from either of the other two groups. Our sample of euthymic patients also included patients with sub‐syndromal depressive symptoms (Tohen et al., 2009). Thus, we did not use a conservative definition of euthymia (Samalin et al., 2016).

For the present study, we restricted our inclusion to euthymic BD‐I patients. Similarly, SZ participants were only included if clinically stable. For both groups, this was done to limit the confounding effect of symptom severity. Most previous work assessing brain activity in response to reward outcome has shown no difference between BD patients and controls (Chase et al., 2013; Nusslock et al., 2012; Smucny et al., 2021), however, reduced brain activity to reward outcomes has been observed in both manic and depressive BD patients in striatal regions (Abler et al., 2008; Redlich et al., 2015). It follows, that the results of BD‐I participants might resemble those of SZ to a greater extent if tested when more symptomatic. Our results show, that during remission, BD‐I patients still show reduced adaptation to reward context, which could be exacerbated during symptomatic episodes and underlie deficits in reward processing observed clinically.

In conclusion, we demonstrate for the first time that patients with BD‐I disorder show adaptive coding deficits, similar to those observed in SZ patients. Adaptive coding in BD appeared more preserved as compared to SZ participants especially in the more anterior part of the right caudate and to a lesser extent also in the right precentral gyrus. Importantly, in both patient groups, adaptation was mostly affected in the small reward context, where patients failed to parametrically increase their response to increasing rewards. These results reinforce the importance of context processing and adaptive coding deficits across mental illnesses (Northoff & Mushiake, 2020; Rosenberg & Sunkara, 2019). Future work will establish whether similar mechanisms are involved in context processing deficits across different disorders.

## DISCLOSURE

All authors report no biomedical financial interests or potential conflicts of interest.

## Supporting information


**Appendix S1** Supporting information

## Data Availability

The data that support the findings of this study are available on request from the corresponding author. The data are not publicly available due to privacy or ethical restrictions
