# Supplementary material for: Comparing adaptive coding of reward in bipolar I disorder and schizophrenia
Source: Hum Brain Mapp. 2022 Sep 16;44(2):523–34. doi: 10.1002/hbm.26078 (PMC9842918; doi:10.1002/hbm.26078)
Supplement: Supplementary file 1 — Appendix S1 Supporting information [file HBM-44-523-s001.docx]

**Supplemental material**

**Acquisition parameters**

Imaging data was collected with a Philips Achieva 3.0T magnetic resonance (MR) scanner using a 32 channel SENSE head coil (Philips, Best, The Netherlands) at the Psychiatric Hospital of the University of Zurich. Functional MRI (fMRI) was acquired in two runs with 195 ascending transverse plane images using a gradient-echo T2*-weighted echo-planar image (EPI) sequence over the whole brain. Acquired in-plane resolution was 3×3mm2, 3mm slice thickness and 0.5mm gap width over a field of view of 240×240mm2, a repetition/echo time (TR/TE) of 2000/25ms and a flip angle of 82°. The first five scans were discarded to account for T1 saturation effects. Slices were aligned with the anterior–posterior commissure. Anatomical data was acquired with an ultrafast gradient echo T1-weighted sequence in 160 sagittal plane slices of 240×240mm2 resulting in 1x1x1mm3 voxels.

**Image preprocessing**

Functional images were corrected for differences in the time of slice acquisition. The Realign and Unwarp functions of SPM8 were used to correct our data for head motion, with an allowed translational head motion limited to ±4 mm. A voxel displacement map, calculated from double phase and magnitude field map data, was used to correct for combined static and dynamic distortions. We performed segmentation, bias correction, and spatial normalization. Finally, images were smoothed using a Gaussian kernel of 6mm width at half-maximum.

**Adaptive coding of reward: Group analysis (including only BD-I patients on antipsychotic medication)**

We performed the group level analyses (i.e. step one: identify reward sensitive ROIs with the [pmod small reward + pmod large reward contrast], step 2: adaptive coding contrast [pmod small reward – pmod large reward]) including only the 17 BD-I patients who were on antipsychotic medication. The results are almost identical to the ones reported in the manuscript: in the more anterior part of the right caudate, SZ group was significantly different from HC (p=0.014) and BD-I patients (p=0.003); in the more posterior part, BD-I patients showed significantly reduced adaptive coding compared to HC (p=0.011), whereas SZ patients only showed a trend for reduction (p=0.065). Both patient groups showed reduced adaptive coding in the right cuneus with respect to HC (SZ p=0.024, BD p=0.013). Finally, for the right precentral gyrus, as before, only SZ patients showed reduced adaptive coding with respect to controls (p=0.038), BD-I patients were neither different from HC (p=0.969, nor from SZ (p=0.13). In addition, a significant difference was also observed for the left caudate (F(2,66)=3.79, p=0.028). Here, only BD-I patients showed reduced adaptive coding with respect to HC (p=0.043), whereas SZ patients showed only trend-level reduction (p=0.074), with no difference between the two patient groups (p=0.87).

**S Table 1.** Whole brain analyses of reward coding regions across all participants (cluster defining threshold p=0.0005)

|  | X | Y | Z | Cluster size | t |
| --- | --- | --- | --- | --- | --- |
| Right precentral gyrus/insula | 60 | 3 | 12 | 250 | 6.77 |
|  | 56 | -3 | 7 |  | 4 |
| Left middle frontal gyrus | -24 | 23 | 54 | 2957 | 6.65 |
|  | -17 | 18 | 61 |  | 6.28 |
|  | -17 | 33 | 54 |  | 5.95 |
| Left angular gyrus | -48 | -60 | 40 | 1814 | 6.09 |
|  | -32 | -61 | 24 |  | 5.15 |
|  | -57 | -39 | 42 |  | 4.96 |
| Left anterior prefrontal cortex | -8 | 54 | -6 | 465 | 6.08 |
|  | -2 | 63 | -2 |  | 4.79 |
|  | -2 | 57 | 9 |  | 4.04 |
| Right superior occipital gyrus | 15 | -88 | 24 | 1582 | 6 |
|  | 15 | -93 | 14 |  | 5.72 |
|  | 3 | -75 | 22 |  | 5.17 |
| Left orbitofrontal cortex | -9 | 24 | -17 | 1443 | 5.75 |
|  | -20 | 5 | 12 |  | 5.68 |
|  | -14 | 9 | 18 |  | 5.35 |
| Left caudate | -20 | -3 | 27 | 471 | 5.49 |
|  | -18 | 6 | 24 |  | 4.44 |
|  | -21 | 14 | 22 |  | 4.3 |
| Right postcentral gyrus | 23 | -28 | 60 | 1266 | 5.47 |
|  | 3 | -31 | 63 |  | 4.65 |
|  | 17 | -33 | 70 |  | 4.42 |
| Right posterior caudate | 21 | -4 | 27 | 574 | 5.43 |
|  | 20 | -15 | 24 |  | 5.3 |
|  | 26 | -31 | 28 |  | 5.26 |
| Right anterior caudate | 17 | 20 | 16 | 145 | 5.12 |
| Left postcentral gyrus | -21 | -31 | 61 | 406 | 5.15 |
|  | -21 | -33 | 76 |  | 4.52 |
|  | -29 | -28 | 66 |  | 4.19 |
| Right postcentral gyrus | 29 | -40 | 63 | 301 | 5.13 |
|  | 27 | -45 | 72 |  | 4.44 |
|  | 32 | -39 | 55 |  | 4.42 |
| Right middle frontal gyrus | 30 | 33 | 13 | 486 | 5.04 |
|  | 9 | 30 | 4 |  | 4.56 |
|  | -3 | 27 | 6 |  | 4.56 |
| Right putamen | 23 | 6 | -11 | 492 | 4.95 |
|  | 23 | 26 | -6 |  | 4.72 |
|  | 24 | 17 | -9 |  | 4.48 |
| Left posterior ventral cingulate cortex | -5 | -49 | 31 | 977 | 4.72 |
|  | 0 | -58 | 25 |  | 4.42 |
|  | -5 | -55 | 16 |  | 4.18 |
| Left thalamus | 0 | -19 | 13 | 251 | 4.56 |
|  | 2 | -3 | 9 |  | 4.05 |
|  | 0 | -31 | 9 |  | 3.92 |

**S Table 2**. Results of one-way Fischer ANOVAs comparing adaptive

coding between HC, SZ and BD-I

| **ROI** | **F** | ***p*** |
| --- | --- | --- |
| Left caudate | 2.85 | 0.064 |
| Left angular gyrus | 2.8 | 0.067 |
| Left anterior prefrontal cortex | 0.006 | 0.99 |
| Left orbitofrontal cortex | 0.059 | 0.94 |
| Left middle frontal gyrus | 2.3 | 0.1 |
| Left postcentral gyrus | 0.88 | 0.42 |
| Left posterior ventral cingulate cortex | 1.06 | 0.35 |
| Left thalamus | 0.28 | 0.76 |
| Right anterior caudate | 6.03 | 0.004 |
| Right posterior caudate | 5.096 | 0.008 |
| Right superior occipital gyrus/cuneus | 6.16 | 0.003 |
| Right middle frontal gyrus | 1.54 | 0.22 |
| Right primary motor area | 0.89 | 0.414 |
| Right postcentral gyrus | 1.38 | 0.26 |
| Right precentral gyrus | 3.29 | 0.043 |
| Right putamen | 0.3 | 0.7 |

**Correlational analyses**

**S Table 3.** HC Group. Correlation between

the adaptive coding contrast and total mount won on the task

| Adaptive coding | Total win |
| --- | --- |
| Right ant caudate | r(p)=-0.07, p=0.74 |
| Right post caudate | r(p)=-0.25, p=0.24 |
| Right precentral/insula | r(p)=-0.17, p=0.41 |
| Right cuneus | r(p)=-0.1, p=0.54 |

| Adaptive coding | PANSS Total | PANSS Positive | PANSS General | BNSS Apathy | CPZ eq | Total win |
| --- | --- | --- | --- | --- | --- | --- |
| Right ant caudate | **r(p)=-0.38, p=0.052** | **r(p)=-0.4, p=0.04** | r(s)=-0.17, p=0.4 | r(p)=-0.2, p=0.33 | r(s)=-0.12, p=0.5 | r(p)=0.22,  p=0.28 |
| Right post caudate | r(p)=-0.21, p=0.29 | r(p)=-0.26, p=0.19 | r(s)=-0.2,  p=0.3 | r(p)=-0.26, p=0.19 | r(s)=0.05, p=0.8 | r(p)=-0.24, p=0.23 |
| Right precentral/insula | r(p)=0.09, p=0.67 | r(p)=-0.15, p=0.45 | r(s)=-0.03, p=0.9 | r(p)=-0.002, p=0.9 | r(s)=0.02, p=0.9 | r(p)=-0.16, p=0.41 |
| Right cuneus | **r(p)=0.41, p=0.036** | r(p)=0.21, p=0.3 | r(s)=0.23, p=0.26 | r(p)=0.23, p=0.26 | r(s)=0.24, p=0.22 | r(p)=-0.11, p=0.59 |

**S Table 4.** SZ Group. Correlational analyses between the adaptive coding contrast, symptom severity and total mount won on the task

**S Table 5.** BD-I Group. Correlational analyses between the adaptive coding contrast, symptom severity and total mount won on the task

| Adaptive coding | PANSS Total | PANSS Positive | PANSS General | BNSS Apathy | CPZ eq | CDS | HAMD | YMRS | Total win |
| --- | --- | --- | --- | --- | --- | --- | --- | --- | --- |
| Right ant caudate | r(p)=-0.29, p=0.18 | r(s)=-0.25, p=0.23 | **r(s)=-0.45, p=0.03** | r(p)=-0.2, p=0.35 | r(p)=-0.06, p=0.8 | r(s)=-0.16, p=0.45 | r(s)=-0.085, p=0.69 | r(s)=0.065, p=0.77 | **r(p)=0.48, p=0.017** |
| Right post caudate | r(p)=-0.13, p=0.55 | r(s)=-0.004, p=0.9 | r(s)=-0.19, p=0.37 | r(p)=-0.23, p=0.29 | r(p)=0.16, p=0.46 | r(s)=-0.1, p=0.64 | r(s)=-0.095, p=0.66 | r(s)=0.19, p=0.37 | r(p)=0.03, p=0.88 |
| Right precentral/insula | r(p)=-0.19, p=0.37 | r(s)=-0.04, p=0.85 | r(s)=-0.31, p=0.15 | r(p)=-0.07, p=0.8 | r(p)=0.015, p=0.9 | r(s)=-0.19, p=0.39 | r(s)=-0.07, p=0.73 | r(s)=0.13, p=0.55 | r(p)=-0.32, p=0.13 |
| Right cuneus | r(p)=-0.25, p=0.25 | r(s)=-0.17, p=0.42 | r(s)=-0.097, p=0.7 | r(p)=-0.17, p=0.42 | **r(p)=0.38, p=0.065** | r(s)=-0.14, p=0.51 | r(s)=-0.21, p=0.33 | r(s)=0.097, p=0.65 | r(p)=-0.25, p=0.25 |
